# General Intelligence

**Published:** 1844-08

**Authors:** 


### GENERAL INTELLIGENCE.

*Medical Schools.*—We have received the Annual Announcements of a number of Medical Institutions. In a number of schools, efforts have been made to increase the strength of the Faculty—the natural result of competition. Want of room forbids any extended notice of the various appointments and changes, in our present number. We notice, among others, the appointment of Leonidas M. Lawson, M. D., editor of the Western Lancet, to the chair of General and Pathological Anatomy and Physiology in the Transylvania University, Lexington. Since his appointment the editor has announced the publication of the Western Lancet simultaneously, in Lexington and Cincinnati.

